# The Continuous Fragility Index of Statistically Significant Findings in Randomized Controlled Trials That Compare Interventions for Anterior Shoulder Instability

**DOI:** 10.1177/03635465231202522

**Published:** 2024-01-23

**Authors:** Mohammed Al-Asadi, Michelle Sherren, Hassaan Abdel Khalik, Timothy Leroux, Olufemi R. Ayeni, Kim Madden, Moin Khan

**Affiliations:** * Faculty of Health Sciences, McMaster University, Hamilton, Ontario, Canada; † St. Joseph’s Healthcare Hamilton, Hamilton, Ontario, Canada; ‡ Division of Orthopaedic Surgery, Department of Surgery, McMaster University, Hamilton, Ontario, Canada; § Division of Orthopaedic Surgery, Department of Surgery, University of Toronto, Toronto, Ontario, Canada; ∥ Department of Health Research Methods, Evidence, and Impact, McMaster University, Hamilton, Ontario, Canada; Investigation performed at McMaster University, Hamilton, Ontario, Canada

**Keywords:** shoulder instability, shoulder general, statistics, fragility index, continuous fragility index, statistical significance

## Abstract

**Background:**

Evidence-based care relies on robust research. The fragility index (FI) is used to assess the robustness of statistically significant findings in randomized controlled trials (RCTs). While the traditional FI is limited to dichotomous outcomes, a novel tool, the continuous fragility index (CFI), allows for the assessment of the robustness of continuous outcomes.

**Purpose:**

To calculate the CFI of statistically significant continuous outcomes in RCTs evaluating interventions for managing anterior shoulder instability (ASI).

**Study Design:**

Meta-analysis; Level of evidence, 2.

**Methods:**

A search was conducted across the MEDLINE, Embase, and CENTRAL databases for RCTs assessing management strategies for ASI from inception to October 6, 2022. Studies that reported a statistically significant difference between study groups in ≥1 continuous outcome were included. The CFI was calculated and applied to all available RCTs reporting interventions for ASI. Multivariable linear regression was performed between the CFI and various study characteristics as predictors.

**Results:**

There were 27 RCTs, with a total of 1846 shoulders, included. The median sample size was 61 shoulders (IQR, 43). The median CFI across 27 RCTs was 8.2 (IQR, 17.2; 95% CI, 3.6-15.4). The median CFI was 7.9 (IQR, 21; 95% CI, 1-22) for 11 studies comparing surgical methods, 22.6 (IQR, 16; 95% CI, 8.2-30.4) for 6 studies comparing nonsurgical reduction interventions, 2.8 for 3 studies comparing immobilization methods, and 2.4 for 3 studies comparing surgical versus nonsurgical interventions. Significantly, 22 of 57 included outcomes (38.6%) from studies with completed follow-up data had a loss to follow-up exceeding their CFI. Multivariable regression demonstrated that there was a statistically significant positive correlation between a trial’s sample size and the CFI of its outcomes (*r* = 0.23 [95% CI, 0.13-0.33]; *P* < .001).

**Conclusion:**

More than a third of continuous outcomes in ASI trials had a CFI less than the reported loss to follow-up. This carries the significant risk of reversing trial findings and should be considered when evaluating available RCT data. We recommend including the FI, CFI, and loss to follow-up in the abstracts of future RCTs.

The shoulder is highly susceptible to instability and dislocations.^1,11^ Numerous trials have been conducted to evaluate various aspects of the surgical and nonsurgical management of anterior shoulder instability (ASI).^5,34,48,54^ While well-conducted randomized controlled trials (RCTs) provide the best available evidence when making treatment decisions, it is important to understand how fragile or robust the results of a trial are when interpreting findings to develop a degree of confidence in the reported estimate of effect.

The traditional threshold of *P* < .05 used in most RCTs for statistical significance has been criticized for being arbitrary and for encouraging a categorical interpretation of evidence.^4,40,41,59^ Several measures have been subsequently developed to supplement the *P* value threshold, most notably, the fragility index (FI), which was first conceptualized by Feinstein^19^ in 1990 and popularized for use in trials by Walsh et al^56^ in 2014. In summary, the FI is the number of events in 1 trial arm that must be changed to nonevents, with the other arm unchanged, so that a statistically significant difference between the 2 trial arms becomes a nonsignificant one. For example, in an outcome with an FI value of 1, it would only take 1 patient’s outcome to change from an event to a nonevent for the findings to no longer be statistically significant. Therefore, fragility can identify significant outcomes relying on only a few patients for significance.

The FI can also provide insight into whether a trial’s loss to follow-up can affect its overall findings.^56^ In trials in which the number of patients lost to follow-up exceeds the FI of the results, the number of patients with an unknown outcome that may possibly be a nonevent is enough to threaten reversing the significance of the trial’s results. Previous FI reviews have reported the percentage of such studies in different fields as ranging from 12.5% to >50%.^32,49,56^ If identified as a widespread issue, this phenomenon can be mitigated by adequately powered studies and accurate estimates of loss to follow-up.

A key limitation of the FI is that it can only be applied to dichotomous outcomes, overlooking vast continuous data that can be equally important in shaping clinical practice. Continuous outcomes include various objective outcomes (eg, range of motion, strength, procedure time) and patient-reported outcomes. Previous reviews that have evaluated the FI report excluding between 25% and 82% of assessed studies because of a lack of dichotomous outcomes.^8,25,51^ Caldwell et al^8^ recently introduced the continuous fragility index (CFI), allowing for the assessment of the fragility of continuous outcomes.

While a recent systematic review assessing the FI of ASI RCTs has been published,^13^ it was limited to dichotomous outcomes such as redislocations, ability to return to play, noncompliance, and need for revision surgery. As such, the purpose of the present review was to evaluate the fragility of significant continuous outcomes in RCTs that compare interventions for ASI to provide readers with an understanding of the quality of available RCT data by which treatment decisions are made.

## Methods

### Search Strategy and Screening

We conducted an electronic search of the MEDLINE, Embase, and CENTRAL databases from inception through October 6, 2022. The search terms included the following: (RCT or randomized) and ((shoulder and (instability or dislocation or subluxation)) or Bankart lesion) (see the Appendix, available in the online version of this article). We automatically removed duplicate records with reference management software (EndNote X9; Clarivate). Using predetermined eligibility criteria, 2 reviewers (M.A. and M.S.) independently screened the title and abstract of each study using systematic review management software (Rayyan 0.9; Qatar Computing Research Institute). We advanced disagreements at the title/abstract review stage to the full-text review stage to prevent any premature exclusion of studies. Discrepancies between the 2 reviewers at the full-text stage were resolved by consulting the senior author (M.K.).

### Eligibility Criteria

The inclusion criteria of this study were as follows: (1) 2-arm RCT assessing the management of ASI; (2) statistically significant finding on ≥1 primary, coprimary, or secondary continuous outcome such as pain, range of motion, questionnaire scores, duration of procedure, and dislocation displacement; (3) details on the sample size, mean values, and spread statistics (standard deviation, confidence interval, range, etc) for the significant outcome(s) for both arms; (4) human study; and (5) English language. The exclusion criteria were as follows: (1) multidirectional or posterior instability studies; (2) unspecified dislocation direction (anterior, posterior, etc); (3) fracture studies; (4) studies investigating perioperative interventions; (5) multiple publications for the same trial with different follow-up times (we included only the latest publication); (6) studies with >2 intervention groups or arms; (7) ASI prevention studies; and (8) letters, reviews, meta-analyses, protocols, biomechanical studies, cadaveric studies, and abstract-only publications.

### Data Extraction

Again, 2 reviewers (M.A. and M.S.) independently extracted relevant information from each study using a collaborative data extraction spreadsheet (Google Sheets; Google) designed a priori. Extracted data included study characteristics (author(s), study design, publication year, etc), patient characteristics (age, sex, etc), intervention, length of follow-up, number of shoulders analyzed in each study group, and number of shoulders lost to follow-up. For each statistically significant continuous outcome in a study such as pain or range of motion, we extracted the sample size, mean values, and spread statistics of the outcome for both study groups. We categorized outcomes as either primary, secondary, or unspecified. In studies that did not specify a primary outcome but conducted a sample size calculation, we considered the outcome used to power the study as the primary outcome and the rest of the outcomes as secondary. We tallied the total number of outcomes, primary outcomes, and secondary outcomes. Overall, 4 of 27 studies (14.8%) did not report loss to follow-up,^10,18,28,45^ which we excluded from calculations involving loss to follow-up. Outcomes were further categorized as either objective (eg, duration of procedure, range of motion) or patient reported (eg, questionnaires, pain measures). We determined the level of evidence for each study by using the classification system set out by Wright et al.^60^

### Calculation of CFI

**Figure 1.**
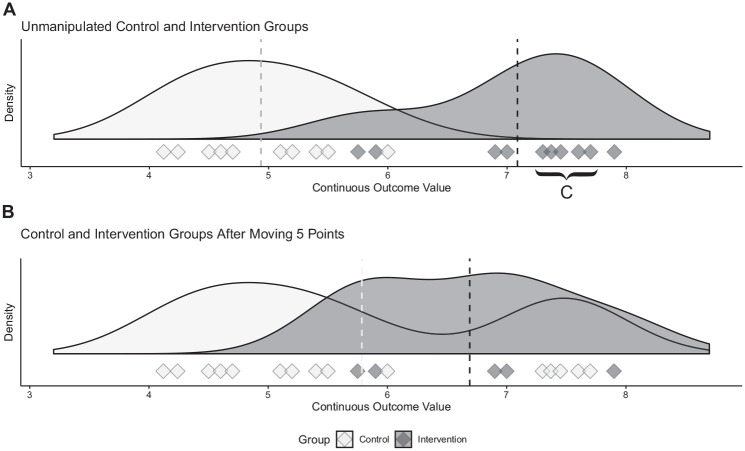
Calculating the continuous fragility index (CFI). The outcomes for 2 theoretical patient groups are illustrated, with each outcome represented as a diamond on the number line. (A) Before any manipulation, there is a statistically significant difference between the groups (*P* < .0001). We first identify the data points closest to and greater than the mean in the intervention group. These data points are moved 1 unit at a time in a sequential manner to the control group until the 2 groups are no longer statistically different (resulting in *B*). In this case, 5 diamonds (*C*) must be moved. Therefore, the robustness of the initial difference can be described with a CFI value of 5.

To calculate the CFI, the full data sets of the measurements of 2 patient groups were required. First, the group with the higher mean was noted. Then, the data point closest to and greater than the mean in the higher-mean group was moved to the lower-mean group. This was repeated until the *P* value obtained from the Welch *t* test no longer demonstrated a statistically significant difference between the 2 groups.^8^ The CFI was defined as the number of data points moved. This CFI calculation is visualized in Figure 1.

The method outlined above requires the full data set of the outcome measure of interest. However, published RCTs often only report summary estimates such as the mean and standard deviation. Therefore, an approximation method has been described by Caldwell et al^8^ that utilizes various descriptive statistics (ie, mean, standard deviation, and sample size) to generate simulated full data sets, assuming a normal distribution, which can then be used to calculate the CFI. To minimize the random error associated with generating random data sets, this model implements multiple iterations that are then averaged accordingly.

All CFI values in this study were calculated using an online calculator that utilized the approximation method described by Caldwell et al.^8,9^ Model inputs included sample size, mean, and standard deviation of both groups in a study. Adjustable model parameters included the number of iterations used to produce the mean CFI, which was set to 5, and the tolerance. The model’s tolerance is the proximity of the random data set’s mean and standard deviation to that of the trial of interest’s respective values; this was set at 0.01.

Overall, 9 included studies used spread statistics other than the standard deviation such as the range, confidence interval, and interquartile range.^∥^ Following Caldwell et al’s^8^ methodology for calculating the CFI, we estimated the standard deviation for those outcomes using appropriate conversion tools.^26,57,58^

### Statistical Analysis

We calculated descriptive statistics across all studies. For a given study with multiple eligible outcomes for analysis, we calculated the CFI of each eligible outcome and averaged them to obtain a study-specific CFI per previous methodology.^16,46^ We also calculated the primary-only study-specific CFI for each study, if applicable, by only considering the fragility of primary and coprimary outcomes. We grouped the included studies based on the type of interventions compared and presented statistics for each. We categorized all outcomes as either objective or patient reported, presented statistics for both, and determined whether there was a statistically significant difference between the 2 outcome types using the Mann-Whitney *U* test. We performed multivariable linear regression in which the outcome variable was the study-specific CFI and the predictor variables were the (1) sample size, (2) journal impact factor, (3) percentage of participants lost to follow-up, (4) duration of follow-up in months, and (5) publication date.

## Results

### Study Selection and Characteristics

**Figure 2.**
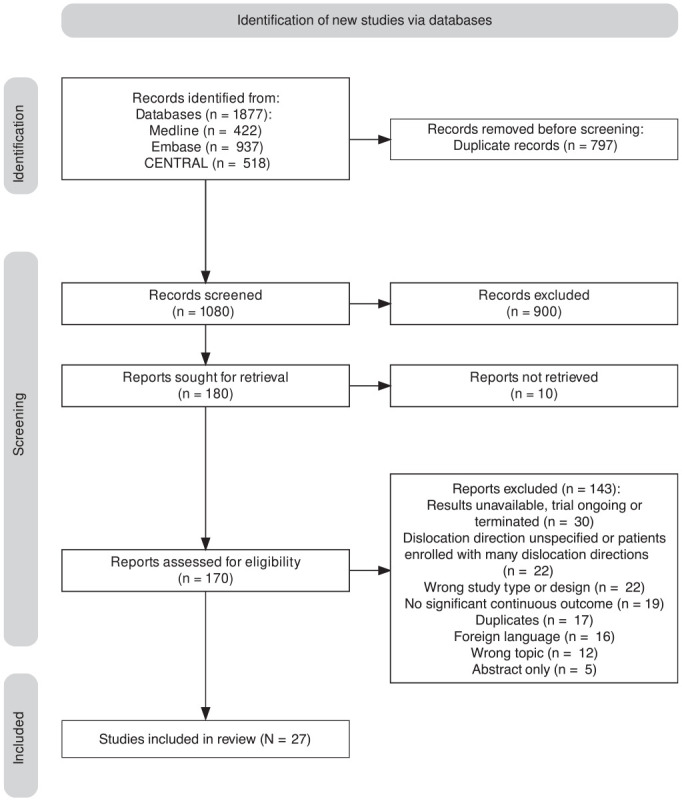
PRISMA (Preferred Reporting Items for Systematic Reviews and Meta-Analyses) flowchart.

We identified a total of 1877 records across the MEDLINE, Embase, and CENTRAL databases (Figure 2), with 1080 remaining for title and abstract screening after removing duplicates. After applying the eligibility criteria, we identified 27 studies for inclusion.^¶^

We also calculated the overall CFI for the following study types (Table 1): (1) studies comparing surgical methods (11 studies),^#^ (2) studies comparing nonsurgical reduction interventions (6 studies),^2,3,21,36,38,52^ (3) studies comparing immobilization methods (3 studies),^24,37,43^ and (4) studies comparing surgical versus nonsurgical interventions (3 studies).^7,35,47^ We grouped the remaining 4 studies into “other.”^17,28,33,39^

**Table 1:** Characteristics and CFI Values of Included Studies^*a*^

					Mean CFI	
First Author (Year)	Comparison	No. of Shoulders Lost to Follow-up	No. of Outcomes^*b*^	Sample Size	All Outcomes	Primary Outcomes
Surgical methods (n = 11)						
Netto^44^ (2012)	ABR vs OBR	8	1	42	0.0	0.0
Bottoni^6^ (2006)	ABR vs OBR	3	1	61	22.0	NA
Castagna^10^ (2009)	AC vs AC and posterior plication	NR	1	40	15.4	NA
Desai^14^ (2021)	ABR with vs without curettage	12	1	80	3.8	NA
Fabbriciani^18^ (2004)	ABR vs OBR	NR	1	60	15.8	NA
Mohtadi^42^ (2014)	ABR vs OBR	34	1	162	27.2	NA
Norlin^45^ (1994)	BR with Mitek anchors vs with bone sutures	NR	1	40	0.0	NA
Salomonsson^53^ (2009)	Putti-Platt procedure vs BR	4	3	66	7.9	7.9
Zarezade^63^ (2014)	Bristow repair vs BR	3	1	37	3.6	NA
Robinson^50^ (2008)	Arthroscopic lavage vs lavage and BR	5	1	83	30.2	NA
Yapp^62^ (2020)	Arthroscopic lavage vs lavage and BR	23	2	65	1.0	NA
Median (IQR)				61 (40)	7.9 (21)	3.4
Nonsurgical reduction interventions (n = 6)						
Akcimen^2^ (2020)	Original vs modified ER reduction	0	1	62	8.2	NA
Amar^3^ (2012)	Milch vs Stimson technique	0	1	60	19.6	NA
Li^36^ (2023)	Modified Milch vs Hippocratic technique	0	10	126	30.4	29.6
Ghane^21^ (2014)	Scapular manipulation vs traction-countertraction	0	3	97	25.7	NA
Maity^38^ (2012)	FARES vs Eachempati method	11	3	149	27.5	33.4
Sahin^52^ (2011)	Scapular manipulation vs Kocher technique	0	1	61	11.4	NA
Median (IQR)				79.5 (65)	22.6 (16)	31.5
Immobilization methods (n = 3)						
Heidari^24^ (2014)	IR vs ER immobilization	5	1	97	8.0	NA
Liavaag^37^ (2009)	IR vs ER immobilization	4	1	51	2.8	2.8
Momenzadeh^43^ (2015)	IR vs ER immobilization	5	1	20	1.2	NA
Median (IQR)				51	2.8	2.8
Surgical vs nonsurgical interventions (n = 3)						
Bottoni^7^ (2002)	ABR vs immobilization	3	2	21	3.6	NA
Kirkley^35^ (2005)	Surgery vs immobilization	9	4	40	2.4	NA
Pougès^47^ (2021)	ABR vs immobilization	2	5	38	0.8	NA
Median (IQR)				38	2.4	NA
Other (n = 4)						
Eshoj^17^ (2020)	Neuromuscular vs standard care exercise	4	6	56	0.6	1
Hurley^28^ (2020)	Latarjet procedure with tranexamic acid vs with placebo	NR	3	100	14.7	15.6
Kim^33^ (2003)	Immobilization vs rehabilitation	0	3	62	8.7	NA
Martinez-Rico^39^ (2018)	Rehabilitation vs rehabilitation with telephone help	1	4	70	9.9	24
Median (IQR)				66 (26)	9.3 (7.6)	19.8 (9.4)
Overall median (IQR)				61 (43)	8.2 (17.2)	11.8 (24.9)

a ABR, arthroscopic Bankart repair; AC, anterior capsulorrhaphy; BR, Bankart repair; CFI, continuous fragility index; ER, external rotation; FARES, fast, reliable, safe; IR, internal rotation; NA, not applicable; NR, not reported; OBR, open Bankart repair.

b Number of significant continuous outcomes that were included in the calculation of the study’s CFI. This included primary, secondary, and unspecified outcomes.

The 27 included studies had a total of 1846 patients (82% male; mean age, 29.4 ± 7.0 years) before any loss to follow-up. The median follow-up time was 24 months (IQR, 30.05). We only included outcomes at final follow-up for analysis. The median sample size was 61 shoulders (IQR, 43). The median percentage of loss to follow-up per study was 6.1% (IQR, 125%), and the median number of shoulders lost to follow-up per study was 5.9 (IQR, 8). All included studies were of level 1 evidence, except 3 studies that were level 2 because of loss to follow-up exceeding 20% of the sample size.^35,43,62^

There was a total of 63 individual outcomes included in the present analysis, of which 17 were primary outcomes, 33 were secondary outcomes, and the rest were unspecified outcomes. We grouped individual outcomes that occurred commonly (≥3 times) across the included studies: Western Ontario Shoulder Instability Index (13 outcomes), pain on a visual analog scale (6 outcomes), reduction time (6 outcomes), and Constant-Murley score (4 outcomes) (Table 2).

**Table 2:** CFI of Individual Outcomes Occurring ≥3 Times^*a*^

	No. of Outcomes	Median	IQR	95% CI
Western Ontario Shoulder Instability Index	13	1.0	2.7	0-3
Visual analog scale	6	13.5	13.8	9.8-33.4
Reduction time	6	26.9	8.4	8.2-56
Constant-Murley score	4	9.5	13.8	0-15.8

a CFI, continuous fragility index.

### Continuous Fragility Index

**Figure 3.**
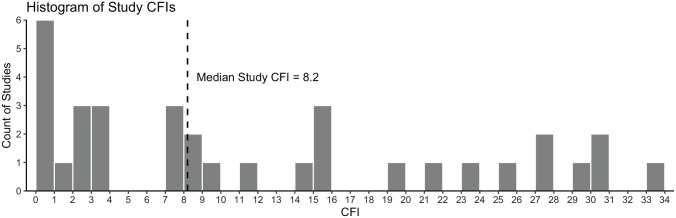
Histogram of study-specific continuous fragility index (CFI) values.

The overall median CFI for included studies was 8.2 (IQR, 17.2; 95% CI, 3.6-15.4; range, 0-30.4) (Table 1). The median primary-only CFI was 11.8 (IQR, 24.9; 95% CI, 1-29.6), although this only involved 8 studies that had an eligible primary outcome. Regarding different study types, the median CFI was 7.9 (IQR, 21; 95% CI, 1-22) for 11 studies comparing surgical methods, 22.6 (IQR, 16; 95% CI, 8.2-30.4) for 6 studies comparing nonsurgical reduction interventions, 2.8 for 3 studies comparing immobilization methods, and 2.4 for 3 studies comparing surgical versus nonsurgical interventions (Table 1). The number of shoulders lost to follow-up was more than the study-specific CFI in 9 studies.^14,17,35,37,42-44,47,62^ Of these, the number of shoulders lost to follow-up was, on average, 6.8 (95% CI, 2.0-11.6) more than the study-specific CFI. A histogram of the frequency of study-specific CFI values is plotted in Figure 3.

The median CFI was 26.9 (IQR, 8.4; 95% CI, 8.2-56) for reduction time outcomes, 13.5 (IQR, 13.8; 95% CI, 9.8-33.4) for visual analog scale outcomes, 9.5 (IQR, 13.8; 95% CI, 0-15.8) for Constant-Murley score outcomes, and 1.0 (IQR, 2.7; 95% CI, 0-3) for Western Ontario Shoulder Instability Index outcomes (Table 2).

We identified 26 objective outcomes, with a median CFI of 14.3 (IQR, 37), and 37 patient-reported outcomes, with a median CFI of 3.6 (IQR, 30). The wide interquartile ranges in CFI values across both groups led to a nonsignificant difference between these outcome types (Mann-Whitney *U* test; *P* = .116).

Collectively, the sample size, journal impact factor, percentage lost to follow-up, follow-up duration in months, and publication date statistically significantly predicted the study-specific CFI in multivariable linear regression (*F*_1,18_ = 17.9; *P* < .001; *R*^2^ = 0.5). Individually, the trial’s sample size was the only statistically significant predictor in the model (*r* = 0.23 [95% CI, 0.13-0.33]; *t* = 4.23; *P* < .001).

## Discussion

The important finding of this study is that the median CFI for RCTs evaluating ASI was 8.2 (IQR, 17.2; 95% CI, 3.6-15.4; range, 0-30.4). In other words, the median number of patients that must be moved from one treatment arm to the other to change a study’s outcome was 8.2. ASI RCTs evaluated here were, on average, comparable with, if not more robust than, available measures of the robustness of the sports orthopaedics literature, which reports a median CFI of 7 (IQR, 11.1).^8,30^ While these summary statistics provide a useful gauge of the robustness of the ASI literature as a whole, the wide ranges and confidence intervals of median CFI values must be noted, indicating that not all study outcomes are equally robust.

Loss to follow-up is an important source of risk in RCTs that can seriously harm a trial’s validity.^15^ The CFI can demonstrate the importance of loss to follow-up by highlighting its ability to affect the statistical significance of outcomes that are fragile enough. In the case of the ASI literature, this study found that 38.6% of included outcomes had a CFI lower than their trial’s loss to follow-up, with the loss to follow-up being, on average, 6.8 (95% CI, 2.0-11.6) shoulders more than the study’s CFI. Therefore, if data from patients lost to follow-up were to be included, there is a risk that the statistical significance of these outcomes may reverse. Compared with our rate of 38.6%, there are 2 previous orthopaedic CFI reviews that report this percentage as 7% and 27.8%, respectively,^22,23^ indicating that this may be a larger problem for the ASI literature. Furthermore, lost data are more likely to be different than available data in the same group of patients,^15^ making this effect more concerning. Overall, this highlights the importance of more robust results that are more resistant to this effect. As well, considering that 20% of orthopaedic trials do not report on loss to follow-up^55^ and that about 15% of studies included in this review did not, it is important that reporting loss to follow-up improves in the future as well as critical focus by authors to maximize patients’ follow-up.

One way to obtain higher CFI values is to increase sample sizes. The CFI algorithm demonstrates a tightly linear relationship between the sample size and CFI if all other variables are held constant.^8^ We also found that sample size was a statistically significant predictor of the study-specific CFI in multivariable regression. Similarly, a statistically significant association between the sample size and study-specific CFI (*P* = .008) was found in Caldwell et al.’s application of the CFI to a previous sports orthopaedics systematic review with a beta coefficient of 0.332.^8,30^ Sample size may also be able to explain differences in the CFI between study types. For example, RCTs comparing surgical arms had a median sample size of 61 (IQR, 40) and a median CFI of 7.9 (IQR, 21), whereas nonsurgical RCTs had a median sample size of 79.5 (IQR, 65) and a median CFI of 22.6 (IQR, 16). Still, it is particularly more difficult to recruit a greater number of patients for surgical RCTs,^20,29^ which comprised approximately 40% of the included studies in this review. One strategy for increasing the sample size in surgical trials is multicenter collaboration.^12^ However, only 1 of the 11 surgical studies included in the present review utilized >1 center in its trial.^63^ Increasing the number of multicenter surgical trials for the management of ASI is an aim for potential future improvement.

In studies using statistical tests less conservative than the Welch *t* test (used in the CFI calculation), a CFI of zero may result. This indicates that simply using the Welch *t* test changed the result to nonsignificant without manipulating the data. In fact, 8 of the 63 included outcomes (12.7%) had a CFI of zero, despite being reported as statistically significant. Ensuring that significance does not hinge on using a less conservative test is another way to improve the robustness of available outcomes reported as statistically significant in the ASI literature.

An FI of 3 has been shown to represent being in the top 25th percentile of statistical robustness across previous trials.^56^ No similar threshold value has been derived for the CFI to date. While the CFI and FI both utilize an iterative process to determine the minimum data modifications required to change statistical significance, their different methodologies render a comparison invalid.^8^ Manipulating dichotomous data has a larger effect on dichotomous tests such as the chi-square test compared with manipulating continuous data sets on tests such as the *t* test.^8^ This leads to generally larger magnitudes of the CFI compared with the FI. For example, a sports orthopaedics systematic review by Khan et al^30^ reported a mean FI of 2, while Caldwell et al’s^8^ analysis of the same studies included in that review produced a significantly greater mean CFI of 9 (*P* < .0001). The present study’s findings further support the importance of complementing the FI with the CFI when possible for an accurate assessment of a study’s statistical robustness.

There have been 5 published studies using the CFI at the time of writing this study, with reported median CFIs of 3, 5, 6, 7 and 9.^8,22,23,27,61^ Therefore, the present review’s median CFI of 8.2 ranks at the higher end of robustness compared with other specialty areas. As an example, Caldwell et al^8^ found a median CFI of 7 for sports orthopaedics RCTs. By this measure, the ASI RCTs evaluated here are, on average, comparable with, if not more robust than, available measures of the robustness of the sports orthopaedics literature. Still a novel metric, studies assessing the CFI in both the orthopaedic and the nonorthopaedic literature will aid researchers in designing robust RCTs that clinicians can have full certainty in. We recommend including the FI, CFI, and loss to follow-up in the abstracts of all future RCTs to allow clinicians to better interpret trial outcomes and evaluate the effect of loss to follow-up on the results.

This study is not without its limitations. First, this study did not have access to outcome data for the included trials. Therefore, the mean, standard deviation, and sample size of each outcome were used to calculate the CFI assuming a normally distributed data set. This may have led to inaccuracies for skewed data sets. The effect was minimized by using multiple iterations (n = 5) when calculating the CFI. Second, not all studies presented the standard deviation, which was required for calculating the CFI. As such, validated conversion tools were used for obtaining the standard deviation required for the CFI calculation.^26,57,58^ Third, this study only considered significant outcomes for inclusion and analysis. However, statistically nonsignificant outcomes also constitute important evidence that can guide clinical practice. Determining their fragility can help identify interventions initially ruled as nonsignificant but fragile, guiding further trials for more definitive evidence. To this end, a reverse FI was first used by Khan et al^31^ to communicate the robustness of a conclusion of nonsignificance, which involves manipulating events until significance is achieved. A reverse CFI for continuous nonsignificant outcomes can also be calculated by modifying the methodology from Caldwell et al,^8^ as described and used by Gupta et al.^23^ Future reviews may focus on this area in the ASI literature.

## Conclusion

More than a third of continuous outcomes in ASI trials had a CFI less than the reported loss to follow-up. This carries the significant risk of reversing trial findings and should be considered when evaluating available RCT data. We recommend including the FI, CFI, and loss to follow-up in the abstracts of all future RCTs.

## Supplemental Material

sj-pdf-1-ajs-10.1177_03635465231202522 – Supplemental material for The Continuous Fragility Index of Statistically Significant Findings in Randomized Controlled Trials That Compare Interventions for Anterior Shoulder Instability Supplemental material, sj-pdf-1-ajs-10.1177_03635465231202522 for The Continuous Fragility Index of Statistically Significant Findings in Randomized Controlled Trials That Compare Interventions for Anterior Shoulder Instability by Mohammed Al-Asadi, Michelle Sherren, Hassaan Abdel Khalik, Timothy Leroux, Olufemi R. Ayeni, Kim Madden and Moin Khan in The American Journal of Sports Medicine

## Footnotes

∥ References 6, 7, 10, 17, 39, 45, 50, 53, 62.

¶ References 2, 3, 6, 7, 10, 14, 17, 18, 21, 24, 28, 33, 35-39, 42-45, 47,50, 52, 53, 62, 63.

# References 6, 10, 14, 18, 42, 44, 45, 50, 53, 62, 63.
