# Supplementary material for: The Continuous Fragility Index of Statistically Significant Findings in Randomized Controlled Trials That Compare Interventions for Anterior Shoulder Instability
Source: Am J Sports Med. 2024 Jan 23;52(10):2667–75. doi: 10.1177/03635465231202522 (PMC11344964; doi:10.1177/03635465231202522)
Supplement: sj-pdf-1-ajs-10.1177_03635465231202522 – Supplemental material for The Continuous Fragility Index of Statistically Significant Findings in Randomized Controlled Trials That Compare Interventions for Anterior Shoulder Instability [file sj-pdf-1-ajs-10.1177_03635465231202522.pdf]

The Continuous Fragility Index of Statistically Significant Findings in Randomized Controlled Trials That Compare Interventions for Anterior Shoulder Instability

Appendix

Search strategy:

Medline

1 exp clinical trials as topic/ or exp Randomized Controlled Trials as Topic/ or exp controlled clinical trial/ or randomized controlled trial.mp. or randomised controlled trial.mp. or controlled clinical trial.mp. or randomly.ab. or randomized.mp. or randomised.mp. or crossover*.mp. or randomized controlled trial.pt. or controlled clinical trial.pt. or trial.ti. 1592412

2 ((shoulder or glenohumeral) adj3 (dislocation or instability or subluxation)).mp. 10043

3 (exp Joint Instability/ and (exp Shoulder/ or exp Shoulder joint/)) or exp Shoulder Dislocation/ or (anterior shoulder instability or glenohumeral instability or bankart).mp. or exp Bankart Lesions/ 9747

4 2 or 3 11254

5 1 and 4 484

6 5 not systematic review.ti. not meta-analysis.ti. not protocol.ti. not letter.ti. 422

Embase

1 exp clinical trial/ or randomized controlled trial.mp. or randomised controlled trial.mp. or controlled clinical trial.mp. or randomly.ab. or randomized.mp. or randomised.mp. or crossover*.mp. or randomized controlled trial.pt. or controlled clinical trial.pt. or trial.ti. 2722658

2 ((shoulder or glenohumeral) adj3 (dislocation or instability or subluxation)).mp. 12025

3 ((exp Joint Instability/ or exp Subluxation/) and (exp Shoulder/ or exp Shoulder joint/)) or exp Shoulder Dislocation/ or (anterior shoulder instability or glenohumeral instability or bankart).mp. or exp Bankart Lesions/ 10753

4 2 or 3 13496

5 1 and 4 1059

6 5 not systematic review.ti. not meta-analysis.ti. not protocol.ti. not letter.ti. 937

Cochrane

ID Search Hits

#1 MeSH descriptor: [Clinical Trial] explode all trees 141

#2 MeSH descriptor: [Randomized Controlled Trial] explode all trees 118

#3 MeSH descriptor: [Controlled Clinical Trial] explode all trees 127

#4 (randomized controlled trial):ti,ab,kw or (randomised controlled trial):ti,ab,kw or (controlled clinical trial):ti,ab,kw or (randomly):ti,ab,kw or (randomized):ti,ab,kw or (randomised):ti,ab,kw or (crossover*):ti,ab,kw 1202172

#5 #1 or #2 or #3 or #4 1202175

#6 ((Shoulder):ti,ab,kw or (glenohumeral):ti,ab,kw) NEAR ((dislocation):ti,ab,kw or (instability):ti,ab,kw or (subluxation):ti,ab,kw)

#7 MeSH descriptor: [Joint Instability] explode all trees

#8 MeSH descriptor: [Shoulder] explode all trees

#9 MeSH descriptor: [Shoulder Joint] explode all trees

#10 #7 and (#8 or #9)

#11 MeSH descriptor: [Shoulder Dislocation] explode all trees

#12 MeSH descriptor: [Bankart Lesions] explode all trees

#13 (anterior shoulder instability):ti,ab,kw or (glenohumeral instability or bankart):ti,ab,kw

#14 #6 or #10 or #11 or #12 or #13

#15 #5 and #14 462

#16 #15 (Word variations have been searched) 518
